# Exploring the Potential Link between Occupational Circadian Rhythm Dysfunction as a Risk Factor for Alzheimer’s Dementia: A novel hypothesis

**DOI:** 10.1002/alz.095676

**Published:** 2025-01-09

**Authors:** Youssef M. Abd El‐Satar, Hazim Mohamed, Youssef A. Ismail, Youssef Haitham, Mohammad Walid

**Affiliations:** ^1^ STEM Neurology & Neuropsychological0 Research Group Egypt (SNRGE), Port Said, Port Said Egypt; ^2^ Faculty of Medicine Cairo Univeristy, Egypt, Cairo, Cairo Egypt; ^3^ Faculty of Medicine Helwan University, Cairo, Helwan Egypt; ^4^ Faculty of Medicine Port Said Univeristy, Egypt, Port Said, Port Said Egypt; ^5^ Faculty of Medicine Suez Canal Univeristy, Egypt, Ismailia, Ismailia Egypt; ^6^ Faculty of Medicine, Arish University, Arish, North Sinai Egypt

## Abstract

**Background:**

The circadian rhythm controls physiological functions across by responding to environmental light cues. fluctuations in this rhythm, such as those induced by irregular work schedules, have been associated with adverse health outcomes, this study aims to assess the increased likelihood risk of developing Alzheimer’s dementia for workers with irregular work schedules.

**Methods:**

Exploring and referencing studies on Alzheimer disease (AD) which had proved an undeniable relationship between dopamine levels with AD onset, this study showcases the relationship of dopamine levels and circadian rhythm and its effect indirectly on Alzheimer as a predisposing factor of AD.

**Discussion and Result:**

Traditionally thought to be the result of neurodegenerative processes, disturbances of the circadian rhythm and sleep‐wake cycle are common symptoms seen in people with AD. However, there may be a possible reciprocal association between circadian abnormalities and the development of AD, suggesting that they may also be risk factors. Circadian rhythms significantly influence dopamine levels throughout the day. This is demonstrated by in vivo studies that show heightened dopamine neuronal firing during the day (7:00‐11:00 h) and at the start of the night (19:00‐23:00 h), with significantly fewer spikes firing in between (11:00‐15:00 h). Furthermore The dopaminergic system has been implicated in the pathogenesis of Alzheimer’s disease and its progression, with patients who suffer from Alzheimer showing low dopamine levels.

**Conclusion:**

Considering the influence of circadian rhythm on dopamine levels, there seems to be a potential interplay between circadian disruptions and neurological health, warranting further investigation into the relationship between circadian rhythm disturbances and the risk of Alzheimer’s dementia onset.

